# Isolation of Oropouche Virus from Febrile Patient, Ecuador

**DOI:** 10.3201/eid2405.171569

**Published:** 2018-05

**Authors:** Emma L. Wise, Steven T. Pullan, Sully Márquez, Verónica Paz, Juan D. Mosquera, Sonia Zapata, Simon K. Jackson, Gyorgy Fejer, Gabriel Trueba, Christopher H. Logue

**Affiliations:** Public Health England, Salisbury, UK (E.L. Wise, S.T. Pullan, C.H. Logue);; Plymouth University, Plymouth, UK (E.L. Wise, S.K Jackson, G. Fejer, C.H. Logue);; Microbiology Institute, Universidad San Francisco de Quito, Quito, Ecuador (S. Márquez, J.D. Mosquera, S. Zapata, G. Trueba, C.H. Logue);; Hospital Delfina Torres de Concha, Esmeraldas, Ecuador (V. Paz)

**Keywords:** Arbovirus, orthobunyavirus, bunyavirus, virus, Oropouche, *Orthobunyaviridae*, *Bunyaviridae*, communicable disease, South America, Ecuador, metagenomic, high-throughput nucleotide sequencing, viruses

## Abstract

We report identification of an Oropouche virus strain in a febrile patient from Ecuador by using metagenomic sequencing and real-time reverse transcription PCR. Virus was isolated from patient serum by using Vero cells. Phylogenetic analysis of the whole-genome sequence showed the virus to be similar to a strain from Peru.

Oropouche virus (OROV) is a negative-sense, single-stranded RNA virus (family *Bunyaviridae*, genus *Orthobunyaviridae*) with a tripartite genome consisting of large (L), medium (M), and small (S) segments. OROV causes a self-limiting acute febrile illness, Oropouche fever (*1*). Since its discovery in Trinidad in 1955 (*2*), >30 outbreaks of OROV have been reported from Brazil, Panama, and Peru, demonstrating the ability of this midgeborne virus to cause epidemics. Approximately 500,000 cases of Oropouche fever have been reported, making OROV one of the most clinically significant orthobunyaviruses (*1*). Two previous studies reported unconfirmed infections in Ecuador by using serologic or antigenic evidence (*3**,**4*). We describe whole-genome sequencing and virus isolation of OROV in Ecuador.

We collected a blood sample from a consenting 41-year-old male patient in Esmeraldas, Ecuador, who sought treatment in April 2016 after 7 days of fever, headache, joint pain, muscle pain, and nausea. The patient reported that he had been in Esmeraldas for >3 months and had not traveled outside the province during that time. RNA was extracted from plasma of the blood sample and tested at Universidad San Francisco de Quito, Ecuador, and Public Health England, UK, for dengue virus (DENV), chikungunya virus (CHIKV), Zika virus, yellow fever virus, Mayaro virus, *Plasmodium *spp.,* Leptospira* spp., and *Rickettsia* spp. by using real-time reverse transcription PCR (rRT-PCR) and conventional RT-PCR assays developed in-house or acquired commercially (Genesig, Primerdesign Ltd., Cambridge, UK). The sample gave borderline results for DENV (quantitation cycle [Cq] 35.3) and CHIKV (Cq 36.6; reference ranges ≤35 positive, 35–40 borderline, >40 negative) and negative results for the other pathogens. 

As an initial screen for other pathogens, we applied unbiased metagenomic sequencing. Analysis of sequencing reads by using Kraken, a system for assigning taxonomic labels to individual reads (*5*), identified 1% reads (5,016 of 464,444) as specific to OROV. We generated an OROV consensus sequence by mapping reads to a reference sequence, which resulted in coverage of 69% for S, 76% for M, and 79% for L OROV viral RNA segments (Technical Appendix). We classified 1,228 reads as DENV serotype 1, all of which mapped to a single 732 nt region of the DENV-1 reference genome. No reads mapped to CHIKV.

After confirmation of the presence of OROV by using a validated rRT-PCR (*6*), we attempted to isolate OROV by using Vero and C6/36 cell lines inoculated with the patient’s plasma. We confirmed virus replication by detecting increasing OROV RNA over time by using rRT-PCR. We obtained whole-genome sequences by sequencing viral RNA from harvested OROV supernatant; each genome segment was sequenced at average depths of coverage of 55,532× for S, 4,954× for M, and 5,674× for L segments. We deposited sequences in GenBank (Technical Appendix). Genetic organization was similar to that of other OROV strains: segment lengths 952 nt for S, 4,387 nt for M, and 6,852 nt for L. 

Phylogenetic analysis (Technical Appendix) showed that the virus we isolated, OROV/EC/Esmeraldas/087/2016, was most closely related to a strain isolated from a patient in Peru during 2008 and excluded the possibility of the virus being a reassortant orthobunyavirus, such as Iquitos virus. This finding suggests a potential introduction across the Peru–Ecuador border; however, further investigation is required to understand the origin and incidence of OROV in Ecuador. The known urban OROV vector, the *Culicoides paraensis *midge*,* is absent in the Pacific Coast region, including Esmeraldas (S. Zapata, pers. comm., 2017 Aug 31), which raises the question of alternative insect vectors in OROV transmission. *Culex* mosquitoes have previously been implicated as vectors in the OROV urban cycle, notably *C. quinquefasciatus* (*1*), a species that is widespread throughout South America (*7*).

DENV and CHIKV rRT-PCR results for this patient were inconclusive. The small proportion of DENV reads in the metagenomic data suggests DENV-1 infection is possible. Using ELISA to detect DENV and CHIKV-specific antibodies may help clarify the results.

It is likely that cases of Oropouche fever go unreported or misdiagnosed. Clinical features of the disease are similar to those of other viral, protozoan, and bacterial diseases previously reported in Ecuador (*1*,*4**,**8**,**9*). OROV might spread unnoticed across a wide geographic area, as suggested by this unexpected detection. Several studies have successfully documented the use of metagenomic sequencing for virus identification in febrile patients (*10*); this approach is becoming more practicable as costs decrease, the major benefit being the ability to detect unexpected or novel viral sequences, as evidenced by this detection of OROV. 

This work highlights the need for increased surveillance of OROV in Ecuador and effective differential diagnostic assays to distinguish between emerging pathogens sharing common clinical descriptions to those already circulating. To clarify the true prevalence of this disease in Ecuador, the OROV rRT-PCR assay will be used to screen archived and newly collected samples from a cohort of patients seeking treatment for acute undifferentiated febrile illness during 2016–2017.

Technical Appendix Isolation process leading to identification of Oropouche virus.
