# Supplementary material for: Isolation of Oropouche Virus from Febrile Patient, Ecuador
Source: Emerg Infect Dis. 2018 May;24(5):935–7. doi: 10.3201/eid2405.171569 (PMC5938787; doi:10.3201/eid2405.171569)
Supplement: Technical Appendix — Isolation process leading to identification of Oropouche virus. [file 17-1569-Techapp-s1.pdf]

Article DOI: https://doi.org/10.3201/eid2405.171569

Isolation of Oropouche Virus from Febrile Patient, Ecuador

Technical Appendix

RT-PCR testing

RNA was extracted from plasma by using the QIAmp viral RNA mini kit (Qiagen, Manchester, UK) and tested for 8 pathogens; Dengue virus (DENV), Chikungunya virus (CHIKV), Zika virus, Yellow fever virus, Mayaro virus, *Plasmodium*, *Leptospira* and *Rickettsia*, by using real-time and conventional RT-PCR assays developed or optimised at PHE, in addition to a commercial multiplex real-time RT-PCR kit (Genesig).

Metagenomic Sequencing

RNA was DNase treated, before cDNA preparation and random amplification by SISPA (Sequence-independent Single Primer Amplification) and Illumina sequencing as described previously (*1*,*2*). An OROV consensus sequence was generated from patient plasma RNA using Quasibam (*3*), following mapping to reference sequences (S segment Genbank accession KP691632, M: KP052851, L: KP691612) using BWA MEM (*4*).

RNA extracted from harvested OROV supernatant was sequenced and analyzed as before. Kraken identified 31% of the reads as specific to OROV. Consensus sequences are deposited in Genbank, strain name 'OROV/EC/Esmeraldas/087/2016', S: MF926352, M: MF926353 and L: MF926354. The S segment contains 2 overlapping open reading frames (ORFs) encoding the nucleoprotein (696 nt) and non-structural protein (276 nt). The M segment contains a single ORF encoding a polyprotein (4,263 nt). The L segment contains a single ORF encoding the RNA-dependant RNA polymerase (6,759 nt). 3′ terminal sequences were confirmed by random amplification of cDNA ends and sanger sequencing (*5*).

OROV/EC/Esmeraldas/087/2016 consensus sequences were used to re-map reads from the patient sample. This improved coverage of the M segment to 98.7%. Sequences from patient

plasma and cultured virus were compared, all segments are >99.8% identical. Eleven positions show single nucleotide polymorphisms, of which 3 are non-synonymous.

Phylogenetic Analysis

For each genome segment, maximum-likelihood phylogenetic trees were generated in MEGA7 by using all complete coding sequences in Genbank. Analysis of S, M, and L segments showed that the most closely related strain was TVP-19256/IQE-7894 from Peru, 2008 (KP795086). Additionally, the Ecuador strain N gene is 97% identical to that of 2 Iquitos virus strains isolated in Peru.

Virus Isolation

A 1:10 dilution of patient plasma was inoculated into Vero and C6/36 cell lines, incubated for 14 days or until cytopathic effect (CPE) was observed. CPE was observed in Vero cells at day 7, no CPE was observed in C6/36 cells. After 1 passage, virus was harvested by low speed centrifugation and filtration of cell-free supernatants.

RNA from supernatants taken at days 5, 7, 11, and 14 was tested by using a modified OROV specific rRT-PCR (*6*). OROV RNA was detected in supernatants from both cell lines and relative quantity of viral RNA increased over time, demonstrating that OROV was replicating within the cultures. Supernatants were also tested for DENV and CHIKV by using virus specific RT-PCR assays. No DENV or CHIKV RNA were detected.

References

1. Atkinson B, Graham V, Miles RW, Lewandowski K, Dowall SD, Pullan ST, et al. Complete genome sequence of Zika virus isolated from semen. Genome Announc. 2016;4:e01116–16. PubMed http://dx.doi.org/10.1128/genomeA.01116-16

2. Greninger AL, Naccache SN, Federman S, Yu G, Mbala P, Bres V, et al. Rapid metagenomic identification of viral pathogens in clinical samples by real-time nanopore sequencing analysis. Genome Med. 2015;7:99. PubMed http://dx.doi.org/10.1186/s13073-015-0220-9

3. Penedos AR, Myers R, Hadef B, Aladin F, Brown KE. Assessment of the utility of whole genome sequencing of measles virus in the characterisation of outbreaks. PLoS One. 2015;10:e0143081. PubMed http://dx.doi.org/10.1371/journal.pone.0143081

4. Li H, Durbin R. Fast and accurate long-read alignment with Burrows-Wheeler transform. Bioinformatics. 2010;26:589–95. PubMed http://dx.doi.org/10.1093/bioinformatics/btp698

5. Acrani GO, Tilston-Lunel NL, Spiegel M, Weidmann M, Dilcher M, Andrade da Silva DE, et al. Establishment of a minigenome system for Oropouche virus reveals the S genome segment to be significantly longer than reported previously. J Gen Virol. 2015;96:513–23. PubMed http://dx.doi.org/10.1099/jgv.0.000005

6. Weidmann M, Rudaz V, Nunes MRT, Vasconcelos PFC, Hufert FT. Rapid detection of human pathogenic orthobunyaviruses. J Clin Microbiol. 2003;41:3299–305. PubMed http://dx.doi.org/10.1128/JCM.41.7.3299-3305.2003
